# Radiotherapy in Fibrodysplasia Ossificans Progressiva: A Case Report and Systematic Review of the Literature

**DOI:** 10.3389/fendo.2020.00006

**Published:** 2020-02-12

**Authors:** Esmée Botman, Jan Coen Netelenbos, Thomas Rustemeyer, Linda J. Schoonmade, Jakko A. Nieuwenhuijzen, Bernd P. Teunissen, Marieke Visser, Pieter Raijmakers, Adriaan A. Lammertsma, Max Dahele, Marelise Eekhoff

**Affiliations:** ^1^Department of Internal Medicine Section Endocrinology, Amsterdam Movement Sciences, Amsterdam Bone Centre, Amsterdam UMC, Vrije Universiteit Amsterdam, Amsterdam, Netherlands; ^2^Department of Dermatology, Amsterdam UMC, University of Amsterdam, Amsterdam, Netherlands; ^3^Medical Library, Vrije Universiteit Amsterdam, Amsterdam, Netherlands; ^4^Department of Urology, Amsterdam UMC, Vrije Universiteit Amsterdam, Amsterdam, Netherlands; ^5^Department of Radiology and Nuclear Medicine, Amsterdam UMC, Vrije Universiteit Amsterdam, Amsterdam, Netherlands; ^6^Department of Neurology, Amsterdam UMC, Vrije Universiteit Amsterdam, Amsterdam, Netherlands; ^7^Department of Radiation Oncology, Amsterdam UMC, Vrije Universiteit Amsterdam, Amsterdam, Netherlands

**Keywords:** fibrodysplasia ossificans progressiva (FOP), radiotherapy, heterotopic ossification (HO), [^18^F]NaF PET/CT, ACVR1 gene mutation

## Abstract

Fibrodysplasia ossificans progressiva (FOP) is an autosomal dominant disease, characterized by the formation of heterotopic ossification (HO) in muscles, ligaments, and tendons. Flare-ups, an inflammatory process that often precedes the formation of HO, can occur spontaneously, but trauma is also a common trigger. It is not known whether radiotherapy, especially in higher doses, might cause sufficient trauma or inflammation to trigger a flare-up and subsequent HO in FOP patients. We report the case of a patient undergoing radiotherapy for the treatment of a 1-cm-wide basal cell carcinoma (BCC) of the lower lip. In addition, we present a systematic review of the available literature. Our patient received 54 Gy in 18 fractions with orthovoltage therapy, resulting in a clinical complete response of the tumor. Six months after treatment, there were no signs of HO either clinically or on [^18^F]NaF PET/CT. The systematic review identified 11 publications describing either radiation treatment in FOP or radiation therapy as a cause of HO in non-FOP patients. Six case reports described the use of radiation in FOP patients for various reasons, including one with a high-dose treatment of a lip BCC using superficial X-ray therapy. The remaining five studies described the use of low-dose radiotherapy to prevent or treat either an FOP flare-up or HO formation. None of these cases showed worsening of disease that could be attributed to the use of radiation therapy. Radiation induced HO in non-FOP patients was rare and occurred in five studies. The largest of these studies suggested that HO was induced after treatment with high doses, resulting in more widespread evidence of tissue damage, potentially being the end result of this damage. In conclusion, available reports suggest no contraindication to radiotherapy in FOP patients; although the number of cases was small, systematic toxicity reports often were not available, and none of the reports described high-dose, high-energy radiation treatment at locations such as muscle and joint regions.

## Introduction

Fibrodysplasia ossificans progressiva (FOP) is an autosomal dominant disorder, which is characterized by heterotopic ossification (HO) in muscle, ligaments, and tendons (1, 2). First ossifications usually develop at the age of 6, often affecting the upper back or neck region. With aging, the formation of HO extends to appendicular regions (3). Often, HO formation is preceded by a flare-up, an inflammatory process of uncertain origin (2, 3). Flare-ups can be provoked by (minor) trauma and infections but can also occur spontaneously (3). Whether radiotherapy can cause sufficient trauma to trigger a flare-up, leading to HO, is unclear. Previously, we have demonstrated that [^18^F]NaF PET can be used to detect activity of disease just prior to the formation or progression of HO (4–6). Intravenously administered labeled sodium fluoride ([^18^F]NaF) binds to newly formed hydroxyapatite and, therefore, can be used to detect osteoblastic activity (7). We previously reported that increased [^18^F]NaF uptake was observed within 1 month of surgery as the first sign of HO recurrence in an FOP patient, confirmed 6 months later with CT (6). If radiotherapy does indeed lead to HO formation, it should be detectable by either increased [^18^F]NaF uptake on PET or the presence of HO at the irradiated site on a follow-up CT.

In this paper, we describe a 67-year-old male patient with FOP, who underwent radiation treatment for a basal cell carcinoma (BCC) of the lower left lip. To place results into context, we then performed a systematic review of the literature to address whether radiotherapy is safe in FOP patients.

## Case Report

A 67-year-old male patient with FOP presented with a 1-cm-wide, progressive lesion of the lower left lip. The patient has the classic variant (R206H) of FOP. The cumulative analog joint involvement scale (CAJIS) score was 25 (8). The patient had not had a flare-up for at least 5 years. However, disease activity was observed at multiple sites on [^18^F]NaF PET/CT performed during annual follow-ups.

A skin biopsy, performed with caution to minimize damage to surrounding tissues, diagnosed an infiltrative BCC. It extended up to the deep biopsy margin (2 mm). Since surgery is known as a trigger for a flare-up, radiation treatment was preferred over surgical excision. Because the patient is wheelchair bound due to FOP, orthovoltage therapy was considered as the most practical method, as he could remain in his wheelchair during treatment. The patient underwent 18 sessions (fractions) of radiotherapy over a period of ~4 weeks, with each fraction delivering a dose of 3 Gy for a total dose of 54 Gy. The BCC showed complete clinical remission after treatment. However, soon after treatment, the patient reported increased difficulty in eating because of decreased mobility of the lower lip. In combination with pre-existing jaw ankyloses, the loss of lip mobility increased the difficulty of eating and drinking. To assess whether these problems were caused by formation of HO in the irradiated area, [^18^F]NaF PET/CT (Gemini TF-64; Philips Medical Systems, Best, Netherlands) was performed. This scan, performed 6 months after completion of radiation therapy, did not show any evidence of HO formation, i.e., no increased tracer uptake in the irradiated area, nor any CT evidence of HO in the treated region. In addition, the radiation therapy did not lead to a significant increase in overall activity of disease throughout the body. Almost 2 years after the irradiation, there was still no sign of HO formation at the irradiated site, confirmed by physical examination.

## Systematic Review

This systematic review was conducted in accordance with the Preferred Reporting Items for Systematic Reviews and Meta-Analysis (PRISMA) statement (www.prisma-statement.org). A comprehensive search was performed in the bibliographic databases PubMed and Embase.com from inception to December 6th, 2018, in collaboration with a medical librarian (LS). Search terms included controlled terms (MesH in PubMed and Emtree in Embase) as well as free text terms. The following terms were used (including synonyms and closely related words) as index terms or free text words: “fibrodysplasia ossificans,” “radiotherapy,” “heterotopic ossification,” and “myositis ossificans.” The search was performed without date or language restrictions. Duplicate articles were excluded. The full search strategies for all databases can be found in the Supplementary Material.

**Figure 1:**
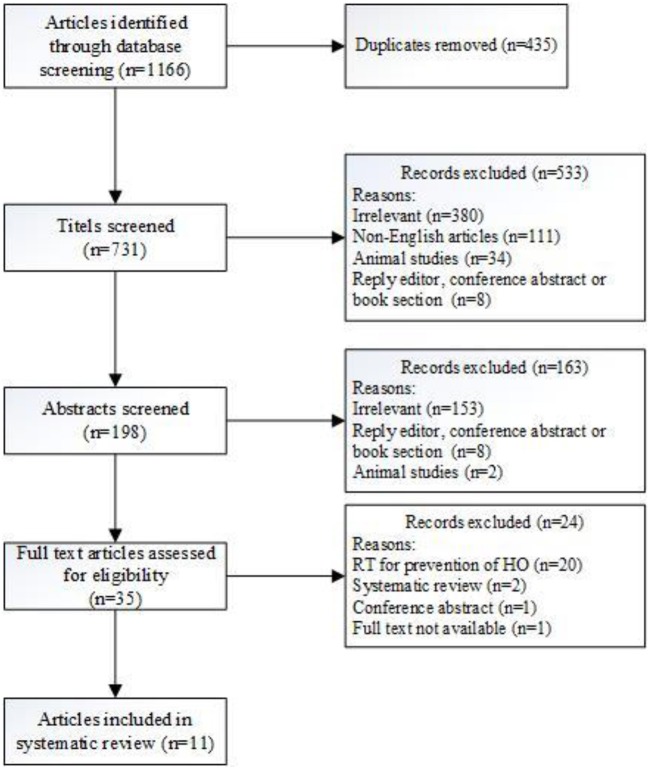
Flowchart of the study selection process.

Using this search strategy, 731 articles were identified. Articles describing radiotherapy in FOP patients or radiation therapy as a (probable) cause of HO were eligible for inclusion (Figure 1). The articles were systematically assessed by two independent reviewers (EB and JCN). Discrepancies were resolved by consensus. After screening titles, abstract, and articles, 11 publications were selected for this systematic review. Of these 11 articles, 6 articles addressed radiotherapy in FOP, and 5 the relationship between irradiation and the formation of HO.

### Radiotherapy in FOP

Not including our own case, radiotherapy in FOP has been described in six other case reports (Table 1). One case reported the radical (high-dose) treatment of a lip BCC using superficial (90 Kv) X-ray therapy (12). The remaining five cases described the use of low-dose radiotherapy to prevent or treat FOP flare-ups or HO formation (9–11, 13, 14). In 4/5 of these cases, a beneficial effect on flare-up symptoms or HO formation was reported (9–11, 14). In 2/5 cases, one or two additional treatment modalities were also reported: a non-steroidal anti-inflammatory drug (NSAID) in both cases and a bisphosphonate in one of them (9, 11). None of the cases reported clinical deterioration or excessive toxicity as a result of radiotherapy (containing) treatment. All but one reported a relatively low dose of radiation (9, 11, 13, 14), consistent with the literature on HO prevention in non-FOP patients (15). Interestingly, Soldic et al. described clinical and radiological benefits after very low doses of fractionated radiotherapy (as low as 2 Gy in two fractions) (14). In the remaining case, which was very similar to ours, a patient received 35 Gy in five fractions on consecutive days for the treatment of a right upper lip BCC. There was a complete response with no evidence of HO at the irradiated site (12). Whether this was confirmed radiographically is not known. In addition, the time interval between radiation therapy and follow-up was not reported. In summary, based on a limited sample of seven patients with FOP (including ours), a range of radiotherapy doses appear to have been well-tolerated, with no reports of excessive or unexpected HO formation and no reports to suggest that the intended outcomes (primarily prevention, treatment of HO, and treatment of BCC) were any worse than expected. However, there was no systematic toxicity reporting, and none of the reports described high-dose, high-energy treatment at specific sites, including muscle and joint regions.

**Table 1:** Articles describing radiotherapy in patients with fibrodysplasia ossificans progressiva.

	**References**	**Age**	**Sex**	**Location**	**Dose (fractions)**	**Indication for RT**	**Follow-up interval after RT**	**Outcome**	**HO formed despite RT-containing treatment?**
1	Benetos et al. (9)	18	♂	Hip	7 Gy (1)	Prevention of post-operative HO, combined with NSAID	1 year	Increased ROM	Yes^a^
2	Dharra et al. (10)	35	♂	Shoulder	10 Gy (5)	Treatment of flare-up	15 months	Relief of symptoms, increased ROM	Unknown
3	Druce et al. (11)	34	♀	Knee	10 Gy (1)	Treatment of flare-up, combined therapy with NSAID and bisphosphonate	2 months	Relief of symptoms.	Yes^b^
4	Frew and Kelly (12)	46	♂	Lip	35 Gy (5)	Basal cell carcinoma	Unknown	Complete response BCC	No
5	Jayasundara et al. (13)	47	♂	Thigh	26 Gy (13)	Prevention of post-operative recurrence of HO	Unknown	Outcome thigh lesion not described	Unknown
6	Soldić et al. (14)	35	♀	Various (*n* = 9) locations	2 (2)−10 Gy (5)^c^	Treatment of ossification after flare-ups	1–10 years	Relieve of symptoms within days, halted progression HO	No

a *Authors state “a small amount of heterotopic bone formed,” suggests less HO than expected*.

b *Amount of HO not quantified, unclear if less than expected*.

c *Also 8 Gy in two fractions, 6 Gy in six fractions, 4 Gy in four fractions, and 3 Gy in three fractions*.

*RT, radiotherapy; HO, heterotopic ossification; Gy, Gray; ROM, range of motion; BCC, basal cell carcinoma; NSAID, non-steroidal anti-inflammatory drug*.

### Development of HO in Non-FOP Patients Treated With Radiotherapy

Five studies were found suggesting that radiation received by non-FOP patients eventually led to HO at the irradiated site (Table 2) (16–20). The interval between actual treatment and formation of HO varied between 1 and 33 years. The largest patient series was from Carl et al. who reported on 15 cases with a range of primary tumors (breast, anal, endometrial, sarcoma, seminoma, bladder, and cervical) (16). Radiation types varied and include cobalt, neutrons, and brachytherapy. Biologically effective doses (for late normal tissue damage, with α/β = 3) ranged from 67 to 214 Gy. However, potential overlap between fields means that local doses may have been higher. HO developed 2–31 years after radiotherapy. Importantly, all patients first developed other signs of tissue damage ranging from plexopathy to ulceration and necrosis as a result of radiation therapy, leading the authors to propose that HO in these patients was an end stage response to the tissue damage caused by radiotherapy. In the other four case reports, neither dose nor tissue damage as a result of treatment was specified (17–20). In three of these cases, no trigger other than radiotherapy for HO was present (18–20). In the remaining case, the authors stated that HO in the ankylosed mandible might have been caused by a combination of factors, including chemotherapy, radiation, prolonged intubation, immobilization, and critical illness neuromyopathy (17).

**Table 2:** Articles describing the formation of heterotopic ossification in non-FOP patients as a late effect of radiotherapy.

	**References**	**Design**	**Number of patients**	**Reason RT**	**Dosage**	**Time interval RT and HO (years)**
1	Carl and Hartmann (16)	Case series	15	Various carcinomas	BED 67–214 Gy^a^	19 (range 2–31)
2	Kruse et al. (17)	Case report	1	Nasopharyngeal carcinoma	Unknown	3^b^
3	Park et al. (18)	Case report	1	Tonsil cancer	Unknown	14
4	Portha et al. (19)	Case report	1	Metastasized mamma carcinoma	Unknown	1
5	Harmon and Nielsen (20)	Case report	1	Testicular tumor	Unknown	33

a *Various kinds of radiotherapy given, potential for overlap could lead to underestimate of radiation dose*.

b *Additional factors: chemotherapy, intubation on intensive care, immobilization, critical illness neuromyopathy*.

*BED, biological effective dose (with α/β = 3 for late tissue effects)*.

## Discussion

To the best of our knowledge, this is the first systematic review of literature relating to the use of radiotherapy in patients with FOP. Including our own case, we found only seven cases in the literature. The available reports suggest that radiotherapy in FOP patients does not lead to the formation of HO at the irradiated site. In addition, there were no reports of excessive or unexpected toxicity and no indication that the intended treatment outcome was poorer than expected. Some caution is required, however, as the number of cases is very small, there was no uniform systematic toxicity reporting or post-radiotherapy assessment, there are limited long-term data, and the effect of high-dose, high-energy radiation to, for example, muscle and joint regions was not described.

One discussion point that can be extracted from these reports is the timing of radiotherapy. Pignolo et al. described that most flare-ups resolved spontaneously within 8 weeks, except those of the hip and back, and of the latter, 75% resolved within 12 weeks (3). One patient was irradiated for a flare-up at the iliopsoas muscle. Radiotherapy was combined with physiotherapy, indomethacin, and disodium etidronate (11). Disodium etidronate, a bisphosphonate, has been used in the past to prevent formation of HO in FOP (21–23), but because of its varying success and side effects, nowadays its use is limited (24). The flare-up was present for 5 weeks prior to treatment. Two months after treatment, it was reported that edema was significantly diminished and pain was relieved (11). Whether this was due to the multi-modality treatment or whether the lesion would have spontaneously resolved is not known with certainty. However, in this case, the patient already had evidence of femoral neurapraxia and neurological deficits at presentation due to the mass. In such a situation, urgent initiation of treatment to avoid permanent nerve damage is important. For milder, non-threatening, flare-ups, a period of observation, to see whether spontaneous regression occurs, would be appropriate. Although apparently effective in the short term, combination treatment did not prevent HO formation, as follow-up CT revealed the presence of calcification at the affected site (11). Unfortunately, the longer-term outcome is not known. Soldic et al. also reported the benefit of radiotherapy in their patient who underwent multiple irradiations at different locations over a prolonged period (14). They used calcification detected on radiographs or CT as a marker of disease. Interestingly, despite low doses of radiation, they reported non-progression of calcification for periods of up to 10 years, and they did not report having to treat previously treated areas again. In the future, it would be interesting to assess disease activity before and after treatment with [^18^F]NaF PET/CT, as this could objectively assess effects of radiotherapy on disease activity (4–6).

The choice between radiotherapy and other treatments need consideration. Treatment of a tumor or prevention/treatment of HO formation both seem reasonable indications based on the literature. The choice between radiotherapy and other modalities will depend on various factors:

1. The risk of secondary tumor induction by radiation, and the effect of radiation on bone.
  A single radiation fraction of, e.g., 7 Gy, as used in myositis ossificans traumatica (MOT) to prevent HO, has only rarely led to a malignancy at the irradiated site (25). Pellegrini et al. hypothesized that this low incidence is due to the already advanced age of most patients developing MOT and the latency period for the malignancy to develop (26). Younger patients have a higher risk of developing a secondary malignancy as a consequence of radiation treatment (27). Even though life expectancy of FOP patients is limited (28), and therefore, the lifetime chance to develop a secondary malignancy due to radiotherapy is also limited, the treatment of a secondary malignancy (e.g., by surgery) is catastrophic for FOP patients.
  Radiation can also have negative effects on bone metabolism, both locally and systemically (29). In addition, FOP patients often underwent multiple glucocorticoid treatments (3), which can also lead to bone toxicity, e.g., reduction in bone mineral density of skeletal bone. Strategies to maximize bone health and mitigate bone toxicity from FOP treatments are required.
2. The potential of either a flare-up or HO formation by alternative therapy (e.g., surgery).
  Although Benetos et al. reported good outcome after surgery followed by indomethacin and radiotherapy (9), traumatic injury is a major trigger for FOP flare-ups and subsequent HO (3, 28). Radiotherapy to prevent HO reoccurrence after surgery is a known and effective strategy in MOT (15, 30, 31), Indomethacin, an NSAID, is known for its post-operative preventative role in MOT (32). Usually, surgery is avoided in FOP because of the effects it can have on disease progression, although resection of HO has been performed to try and improve function, and surgery may also be necessary in certain urgent conditions. If surgery is required, post-operative radiotherapy and/or NSAID treatment to prevent HO formation should be considered.
3. Patient tolerance or risk of non-radiotherapy side effects.
  Glucocorticoids are commonly used for the treatment of flare-ups because of their anti-inflammatory effect. Although their effect on prevention of flare-ups and HO formation has never been rigorously tested, about half of the patients report an improvement in flare-up symptoms when treated with glucocorticoids (3). However, known side effects are, among others, weight gain, proximal myopathy, glucose intolerance, suppression of endogenous hormones, and gastrointestinal toxicity (33). There is extensive experience with NSAIDs in FOP patients (24). About one-third of patients use NSAIDs for flare-ups, although they can lead to gastrointestinal issues and renal toxicity (3). Radiotherapy should not be seen as a replacement for anti-inflammatory drugs but, rather, as a complementary treatment strategy to be considered in certain clinical situations and for selected patients.

Even though radiotherapy seems safe in FOP patients, one should keep in mind that post-irradiation tissue damage (e.g., fibrosis) leading to (even minimal) mobility/function loss can have a significant impact on the quality of life of patients. Patients are highly dependent on their remaining function, and any disturbance can significantly affect daily life. Any intervention, including radiotherapy, should take this into account, and where possible, risks should be kept as low as possible.

In conclusion, the risk of HO induction by radiation in non-FOP patients is, as demonstrated by the few cases in our systematic review, very small and usually part of more widespread tissue damage. Based on available literature, radiotherapy-induced HO formation does not seem to be a problem in non-FOP or FOP patients. As follow-up data are limited, radiotherapy for FOP patients should only be considered in specific situations, e.g., post-operatively after surgery or to reduce flare-up edema when causing neurological deficits. As [^18^F]NaF is the only *in vivo* disease activity marker currently available, pre-treatment and follow-up imaging using [^18^F]NaF PET/CT should be considered to evaluate the effects of interventions, including radiation, on local, and systemic FOP activity.

## Data Availability Statement

All datasets generated for this study are included in the article/Supplementary Material.

## Ethics Statement

The studies involving human participants were reviewed and approved by Medical Ethical Review Board, Amsterdam UMC, Vrije Universiteit Amsterdam, Netherlands. The patient/participants provided their written informed consent to participate in this study. Written informed consent was obtained from the individual for the publication of any potentially identifiable images or data included in this article.

## Author Contributions

EB, ME, JAN, MD, and TR: study design, data analysis, data interpretation, and drafting manuscript. EB, ME, JCN, MD, and TR: study conduct. EB, ME, JCN, and LS: data collection. PR, BT, AL, MV, and JAN: revising manuscript content. EB, ME, JAN, MD, TR, AL, BT, PR, MV, LS, and JAN: approving final version of manuscript. EB, ME, and MD: taking responsibility for the integrity of the data analysis.

### Conflict of Interest

The authors declare that the research was conducted in the absence of any commercial or financial relationships that could be construed as a potential conflict of interest.
