# Supplementary material for: Radiotherapy in Fibrodysplasia Ossificans Progressiva: A Case Report and Systematic Review of the Literature
Source: Front Endocrinol (Lausanne). 2020 Feb 12;11:6. doi: 10.3389/fendo.2020.00006 (PMC7028822; doi:10.3389/fendo.2020.00006)
Supplement: Supplementary file 1 [file Data_Sheet_1.pdf]

Supplementary Material

1.1.1 PubMed History December 6, 2018

Search	PubMed Query – December 6, 2018	Items found
#3	#1 AND #2	515
#2	"Radiotherapy"[Mesh] OR "radiotherapy"[Subheading] OR radiotherap*[tiab] OR radiati*[tiab] OR irradiati*[tiab] OR x ray therap*[tiab]	673905
#1	"Myositis Ossificans"[Mesh] OR "Ossification, Heterotopic"[Mesh] OR fibrodysplasia ossifican*[tiab] OR myositis ossifican*[tiab] OR ossifying myositis[tiab] OR ossifying fibrodysplasia [tiab] OR stone man[tiab] OR acvr1[tiab] OR fop[tiab] OR heterotopic ossifica*[tiab] OR ectopic ossifica*[tiab] OR pathologic ossifica*[tiab]	12684

1.1.2 Embase.com History December 6, 2018

Search	Embase.com Query – December 6, 2018	Items found
#3	#1 AND #2	651
#2	"radiotherapy'/exp OR radiotherap*:ab,ti,kw OR radiati*:ab,ti,kw OR irradiati*:ab,ti,kw OR (x-ray NEAR/3 therap*):ab,ti,kw	934801
#1	'ossifying myositis'/exp OR 'heterotopic ossification'/exp OR (fibrodysplasia NEAR/3 ossifican*):ab,ti,kw OR (myositis NEAR/3 ossifican*):ab,ti,kw OR (ossifying NEAR/3 myositis):ab,ti,kw OR (ossifying NEAR/3 fibrodysplasia):ab,ti,kw OR ‘stone man’:ab,ti,kw OR acvr1:ab,ti,kw OR fop:ab,ti,kw OR (heterotopic NEAR/3 ossifica*):ab,ti,kw OR (ectopic NEAR/3 ossifica*):ab,ti,kw OR (pathologic NEAR/3 ossifica*):ab,ti,kw	12143
